# Interactive voice response technology for symptom monitoring and as an adjunct to the treatment of chronic pain

**DOI:** 10.1007/s13142-012-0115-x

**Published:** 2012-02-01

**Authors:** Gregory Lieberman, Magdalena R Naylor

**Affiliations:** Department of Psychiatry, Clinical Neuroscience Research Unit, University of Vermont College of Medicine, 1 South Prospect Street, UHC, Burlington, VT 05401 USA

**Keywords:** Interactive voice response, Chronic pain, Cognitive behavioral therapy, Coping, Symptom monitoring

## Abstract

Chronic pain is a medical condition that severely decreases the quality of life for those who struggle to cope with it. Interactive voice response (IVR) technology has the ability to track symptoms and disease progression, to investigate the relationships between symptom patterns and clinical outcomes, to assess the efficacy of ongoing treatments, and to directly serve as an adjunct to therapeutic treatment for chronic pain. While many approaches exist toward the management of chronic pain, all have their pitfalls and none work universally. Cognitive behavioral therapy (CBT) is one approach that has been shown to be fairly effective, and therapeutic interactive voice response technology provides a convenient and easy-to-use means of extending the therapeutic gains of CBT long after patients have discontinued clinical visitations. This review summarizes the advantages and disadvantages of IVR technology, provides evidence for the efficacy of the method in monitoring and managing chronic pain, and addresses potential future directions that the technology may take as a therapeutic intervention in its own right.

## INTRODUCTION

During the past two decades, there has been considerable growth in the use of computer-mediated technologies for assessment and treatment. One such technology is interactive voice response (IVR), an automated telephone-based interface that provides patients and research subjects with direct access to pre-recorded questionnaires, educational materials, custom messages, and even therapeutic assistance [1]. Typically, patients or research subjects are provided with a phone number to connect to a computer database interface that provides high-quality pre-recorded interactions. Using a branched logic format, IVR menus are navigated and questions are answered by pressing specific buttons on a touch-tone keypad that correspond to different options or responses. These selections are then stored for later analysis. Additionally, some IVR systems provide patients and subjects with the ability to record their own verbal responses to open-ended questions. Many modern applications of IVR now utilize voice recognition software rather than touch-tone responding for increased convenience. The continued success of IVR as a clinical and research method is due in part to improvements in voice recognition software and in part to development of open-standard IVR platforms such as the World Wide Web Consortium’s Voice Extensible Markup Language and the Speech Application Language Tags Forum [2]. There are also several companies (e.g., TeleSage, Chapel Hill, NC and DirectConnect, Omaha, NE) that provide access to established IVR systems.

Some of the simplest and most common applications of IVR to clinical research are participant screening, study enrollment, and group randomization [3, 4]. Computer algorithms can be used to analyze subjects’ responses to demographic questions (e.g., age and gender) in order to determine study eligibility and to randomly assign participants to different experimental conditions. Some other established applications of IVR include survey/questionnaire administration [5–7]; tracking of medication adherence [8, 9]; smoking cessation education and counseling [10, 11]; alcohol and substance abuse withdrawal and relapse tracking [12–15]; monitoring of drinking [16–19], smoking [20], and binge eating behaviors [21]; screening for and monitoring symptoms of depression [22–25], anxiety [23], and OCD [26, 27]; reporting on the efficacy and side effects of pharmaceutical regimens [9, 25, 28]; symptom monitoring in cardiac patients [29]; and psychological performance assessment [30].

In addition to passively using IVR to monitor ongoing medical conditions, IVR is also applied as an adjunct to medical treatment. For example, IVR has been employed to assist with weight loss and cholesterol management [31], to facilitate healthier lifestyles in a pre-diabetic population [32], to help monitor and improve glucose levels in patients with diabetes [33], to teach and facilitate stress management techniques [34], to improve patient safety after hospital discharge [35], to provide brief intervention for alcohol abuse [13, 36–38], to assist with smoking cessation [39], and for clinical interventions such as self-administered psychotherapy for depression [40] and relapse prevention following cognitive behavioral therapy (CBT) for alcohol use [41].

IVR has proved to be an extremely effective tool in the study of chronic pain, as well. Most patients with chronic pain experience periods of fluctuating symptom severity. Due to this variability, IVR can be a powerful monitoring tool in that patients are able to report symptom severity on an hourly, daily, weekly, or real-time event-contingent basis, allowing clinicians and researchers to control or, if utilized in a properly designed study, eliminate the influence of recall bias on perception of symptom severity. IVR is also useful for keeping track of medication need and adherence, efficacy and adverse side effects of medications, and effectiveness of ongoing therapeutic regimens. Overall, it has been clearly demonstrated that inclusion of IVR in chronic disease management has the potential to improve clinical outcomes while decreasing treatment-associated costs [42, 43]. This review will focus specifically on applications of IVR to the study and treatment of chronic pain, though its applicability to the study and treatment of other psychological disorders is also suggested by the current literature.

## ADVANTAGES OF IVR IN CLINICAL RESEARCH

The major appeals of using IVR in clinical research are its convenience of remote access, high level of accessibility, and cost-effectiveness [44]. Telephones are simple to use and familiar to people of most demographics, an advantage of IVR over some of the similar internet-based approaches that are also currently being employed. IVR systems are available 24 h per day, are accessible by multiple patients at the same time, can utilize different languages for survey administration, and are capable of reaching broad populations. IVR is easier to access than in-person interviews for populations with socioeconomic concerns that may rule out easy computer access, might have difficulty with the unfamiliarity of computer-based approaches, or live too far away to realistically visit the research center on a regular basis. IVR systems can also be used to initiate calls, text messages, or even emails to act as reminders for those who have not called in by a certain prearranged time.

Unlike traditional telephone interviews and written questionnaires, IVR systems are capable of collecting information and immediately storing it in a computer database without the need for labor-intensive clinician or researcher involvement [45], even for extremely large numbers of study participants. Research has demonstrated that IVR interviews are as valid as in-person written questionnaires, live telephone interviews, and internet-based approaches when using measures such as symptom-quantifying questionnaires [6, 7, 12, 45–49].

One of the few difference observed between symptom reporting via IVR and in-person interviews is that some groups of subjects report an increased sense of anonymity that allows them to honestly disclose sensitive information to an automated system that they might be too embarrassed or otherwise unwilling to discuss face-to-face or via a written questionnaire [2, 12, 23, 50–53]. For example, studies show that subjects report higher levels of drug and alcohol abuse when using IVR as compared to live interviews [18, 37, 49, 54].

Compared to in-person and live telephone surveys, IVR ensures consistent phrasing, pacing, and intonation of questions being asked, and negates any interviewer bias that might otherwise exist [2]. IVR can also present questionnaires to multiple participants at the same time, does not depend on participant literacy, and can utilize as many different languages as are needed. Hundreds or thousands of calls can be processed in a single day [55]. In general, participants in IVR studies report that the experience is both easy and convenient [27, 42, 56, 57]. Up to 85% of participants reported satisfaction with using an IVR system, 82% believed that IVR should accompany routine health care, 76% would choose to receive automated calls in the future, and only 16% were bothered by receiving automated IVR calls/reminders [42, 43, 58].

Research has shown that levels of IVR compliance tend to be relatively high. In two longitudinal studies utilizing daily reporting, participants placed calls more than two out of every 3 days when reporting on the severity of pain symptoms and medication usage [59], as well as to enhance brief alcohol intervention (91% median call rate) [13]. In one study, one fourth of diabetes patients completed as many as 91% of daily calls [43]. In another experiment, more than 80% of participants with chronic pain completed 50% or more of daily calls [60]. In a research setting, compliance can be further improved by offering incentives for placing on-time calls to the IVR system, or for consecutive daily/weekly calls. Combination of daily incentives with bonuses for completing consecutive calls on all 7 days of a calendar week resulted in a total data capture rate of 98.8% (93.8% on-time) [54]. Within a population of recovering alcohol and drug users, IVR compliance levels were higher than completion rates for written daily questionnaires [12]. Daily pain reporting using electronic diaries [61] appears to have comparable levels of compliance (94%) to IVR, as well. Unlike pencil-and-paper and electronic diaries, however, IVR also has the ability to track patient compliance in real time and bring compliance concerns to the attention of researchers before they become too severe [55].

One of the most useful advantages of IVR systems is that they allow subjects to easily place reports in an event-contingent, real-time basis (e.g., immediately after a change in symptom severity) or according to a specific schedule, both of which have advantages over reports placed retrospectively. These momentary and daily assessments result in more accurate reports than retrospective assessments, possibly by reducing or eliminating recall biases [61–66]. Possible explanations for this effect are that patients might retrospectively fail to consider pain-free periods when reporting average pain levels [64], place disproportionate weight on peak or more recent symptom experiences [67], employ various cognitive heuristics, or even just have difficulty remembering all pertinent events over a longer reporting period [67]. A summary of IVR’s advantages and disadvantages is presented in Table 1.

**Table 1:** Advantages and disadvantages of IVR in clinical research

Advantages	Disadvantages
Easy 24/7 system access from most locations	Unreliable telephone access in some areas
Cost-effectiveness	Survey interruption due to dropped calls
Familiarity with telephone technology	Not all instruments/surveys are validated for IVR
High level of compliance	Inability to answer participant questions in real time
Increased perceived anonymity	Inability of patients to seek clarification during survey
Ability to collect real-time momentary assessments	Less personal than meeting with a clinician
More accurate than retrospective reporting	Requires script validation, piloting, participant training
Consistency of survey administration	Requires staff to program and maintain IVR system
Not dependent on patient literacy	Costs associated with setup or commercial hosting
Not dependent on patient computer skills	Limited to auditory presentation (lack of visuals)
Allows for a large numbers of participants	
Allows simultaneous access to multiple patients	
Efficient data collection and storage	
Efficient tracking of recruitment and compliance	
Inclusion of automated reminders	

Table adapted from Abu-Hasaballah et al. [2]

## DISADVANTAGES OF IVR IN CLINICAL RESEARCH

Some disadvantages of IVR in research include the initial time and financial commitments required to write scripts, develop recordings, and acquire the necessary hardware and software to run an IVR system, as well to maintain equipment and back-up data over time [50]. However, the up-front costs of programming and hardware acquisition are fixed costs that can be spread out across an unlimited number of participants. Alternatively, it is possible to contract IVR setup and maintenance to one of several companies that offer this service, but this option represents a significant financial commitment, as well. While the IVR hosting costs are not fixed, the incremental cost of adding new participants is quite small. Whichever option is employed, steps to prevent loss of data (e.g., due to computer downtime, power outage, phone service interruption, etc.) must be taken. It is also imperative that stored IVR data be maintained just as securely as any other patient records.

Another major disadvantage of IVR is that some participants of low socioeconomic status or who live in remote regions may not have regular access to telephone service. There is evidence that cellular phone use is prevalent enough even in developing countries [68] to support over-the-phone interventions as viable models, but subjects who participate in IVR studies using cellular phones run the risk of poor reception and dropped calls. Most studies address these concerns by utilizing toll-free phone numbers that participants can use to access the IVR system for free and allowing IVR computers to pause a session when a call is disconnected, continuing the session at the same location when the participant reconnects.

While IVR compliance rates are high, they are not perfect. Sophisticated statistical techniques such as multi-level analyses [69, 70] are required for interpretation of within-subject causal relationships since the data tend to be highly correlated. If such analyses are planned, studies should incorporate reminder calls and incentives for compliance. Although the available software can analyze data with missing days, the underlying assumption is that behaviors are the same on non-reporting days as on days when calls are made. Another approach is to analyze “dyads,” or blocks of two consecutive days upon which daily calls are placed [70].

Another concern in IVR use for symptom monitoring is reactivity: high-frequency reporting might actually influence the severity of symptoms being reported. For example, some concern exists that frequent reporting of symptoms might result in rumination/catastrophizing regarding those symptoms, thereby increasing their severity [63], though other reports suggest that this is might not be the case [61, 64]. The possibility of “stereotypic responding,” or habitually responding in the same manner rather than accurately assessing and reporting symptom states at the time of reporting, should also be considered [63]. It must also be kept in mind that the validity and effectiveness of only a limited number of questionnaires, surveys, and therapeutic approaches have been tested using IVR, to date. Adaptation of new surveys to IVR systems may require script validation and extensive piloting [2], and it is possible that not all methods will maintain validity when employed via IVR.

One proposed weakness of IVR is the lack of personal interaction that comes with in-person interviews. Though this can actually be considered a strength when collecting sensitive information about socially unacceptable behaviors, depressed research subjects reported a stronger ability to describe their feelings during in-person interviews and rated interview experiences with clinicians higher than IVR [23]. While clinicians are able to tailor personal feedback for individual patients using IVR, this may not be as effective as using in-the-moment prompts to delve deeper into issues as they arise. While many IVR systems let patients listen to questions more than once, they do not allow patients to seek clarification regarding survey items or ask questions of their own as they would do during an in-person interview [2, 55]. Despite these disadvantages, however, IVR could be an extremely useful and cost-effective method for many experimental and clinical approaches. Perhaps the most proven application of IVR is for longitudinal tracking of symptom progression in a regular, reliable manner.

## IVR FOR SYMPTOM MONITORING IN CHRONIC PAIN

Most patients experience fluctuations in pain severity that last hours, days, weeks, or months. IVR technology allows patients to monitor and report the severity of their symptoms as frequently as desired or in real time on an event-contingent basis (e.g., when migraine symptoms begin). Daily data concerning pain severity, negative affective components of pain, and the deleterious effects of chronic pain on day-to-day life can be monitored to create a bigger picture of the progression of the disorder on a short-term and longitudinal basis.

IVR has been utilized to track changes in the severity of pain symptoms and treatment outcomes in patients with both short-term [45] and chronic pain [44, 71, 72]. The results of many questionnaires that assess pain severity via IVR are not statistically different from those obtained using traditional live telephone interviews [45] and in-person evaluations [47]. Validity of daily IVR reports has also been compared to retrospective reports covering previous 1, 3, 7, and 28 days [67]. It has also been suggested that, for chronic pain in particular, specific processes that occur within a given day likely have stronger effects on symptom severity than processes that occur across multiple days [69, 73]. Retrospective reports of pain severity tend to be higher than real-time daily reports, suggesting that daily IVR reporting is more accurate than recalled ratings reported retrospectively [67]. For example, daily reports of pain severity averaged over the course of a week are typically higher than weekly reports of pain severity [55]. It has also been shown that IVR’s accuracy is not entirely dependent on real-time, event-contingent reporting: end-of-day IVR ratings of pain severity do accurately reflect real-time reports provided throughout that day [62]. This suggests that once-daily IVR symptom reports are an accurate measure of chronic pain progression.

IVR is also a useful tool for tracking changes in symptom presentation over extended periods of time [45, 74, 75]. IVR is especially useful in the study of chronic pain because of its ability to monitor not only pain severity, but also comorbid symptoms such as depression, anxiety, and quality and quantity of sleep [70, 76]. Many of the emotional symptoms that present comorbidly with chronic pain can be assessed by adapting established questionnaires (e.g., the Hamilton Depression Rating Scale [77]) for IVR use. Examination of the relationship between negative emotions (anger, sadness, and stress) and pain variables using IVR has revealed significant correlations between emotion variables, pain severity, and ability to control pain [60]. Because these relationships exist, it is important that IVR systems that track depressive systems be programmed to notify study personnel if a subject reports an increased incidence of dangerous thoughts or behaviors. Depending on patient responses, follow-up interventions cannot only be arranged when necessary, but also custom-tailored for individual patients [78].

These findings are important because they provide insight into the relationships between negative emotions and pain, and suggest that therapeutic interventions for chronic pain might be effective in reducing both pain severity and negative emotions. The novel use of IVR to perform accurate symptom monitoring may extend the gains of chronic pain treatment by providing regular and/or real-time assessments of the chronic pain progression and its relationship to mood, stress, and coping. Taken one step farther, IVR is also a useful tool for gauging the efficacy of ongoing treatments.

## IVR FOR ASSESSMENT OF TREATMENT EFFICACY IN CHRONIC PAIN

As useful a tool as IVR is for monitoring symptom status and pain progression under normal daily circumstances, it is also a helpful tool for quantifying the effectiveness of ongoing treatments for chronic pain. IVR is utilized during pharmaceutical trials in order to track pain symptoms, negative emotions associated with pain, and adverse side effects of specific medications [9, 79–81]. Some studies merely examine symptoms and side effects longitudinally, while others take advantage of IVR’s ability to provide regular and/or real-time data in order to examine time of pain onset, time for symptoms to fade after medication administration, satisfaction with pain control, and patterns of associated negative emotions and quality of sleep [82, 83]. Further, examination of the relationships between symptom progression, negative affect, type of therapeutic approach, and the level of success of treatment may offer insight into why some treatments work for some patients but not others, or even assist with deciding which treatment options to pursue for specific patients.

The effectiveness of therapeutic interventions such cognitive behavioral therapy and coping skills training for treating chronic pain disorders has been known for some time [73, 84–92]. More recently, IVR has also been used as a follow-up to treatment in order to assess the efficacy of therapeutic interventions. This data stresses the importance of coping skills and control of catastrophizing to successful treatment of chronic pain [93, 94]. Patients who received eight sessions of pain coping skills training reported significantly decreased levels of pain severity and degree of catastrophizing 2 months after surgical intervention for pain [94]. IVR provides an easy and convenient way to track these symptoms and quality-of-life concerns longitudinally, both during and after the completion of therapeutic intervention.

## THERAPEUTIC IVR FOR EXTENDING TREATMENT GAINS IN CHRONIC PAIN

Patient use of CBT skills over time tends to decline once CBT has ceased, and treatment gains accomplished by CBT are not maintained if patients stop using coping skills [44, 95–97]. If patients continue to use and practice these skills, the therapeutic effect is not only maintained but is augmented [44, 98]. The creation of automated IVR systems that not only monitor symptoms, but also guide patients through reminders and practice sessions of the coping skills learned in CBT represent a convenient and inexpensive method that may greatly contribute to extending therapeutic treatment gains [44, 71]. The therapeutic interactive voice response (TIVR) program developed by Naylor et al. is a telephone interface that is capable of collecting data from patients just as standard IVR systems do, but also provides therapeutic benefits that persist long past the actual duration of psychotherapeutic intervention [44, 71]. One experiment followed 11 weekly 90-min sessions of CBT with 4 months of daily TIVR that utilized a relapse prevention model of behavioral change [59, 99] to decrease pain severity and improve coping. Many of the coping skills that are taught to patients [59] with chronic musculoskeletal pain as part of a CBT regimen [85] can be easily converted into TIVR review and practice sessions [44]. These coping skills have been shown to be most successful when practiced regularly outside of the clinical setting. To extend therapeutic gains of group CBT, TIVR has been effectively applied to the treatment of chronic pain by reinforcing pain coping skills and providing patients with educational support that can be accessed on-demand.

**Fig 1:**
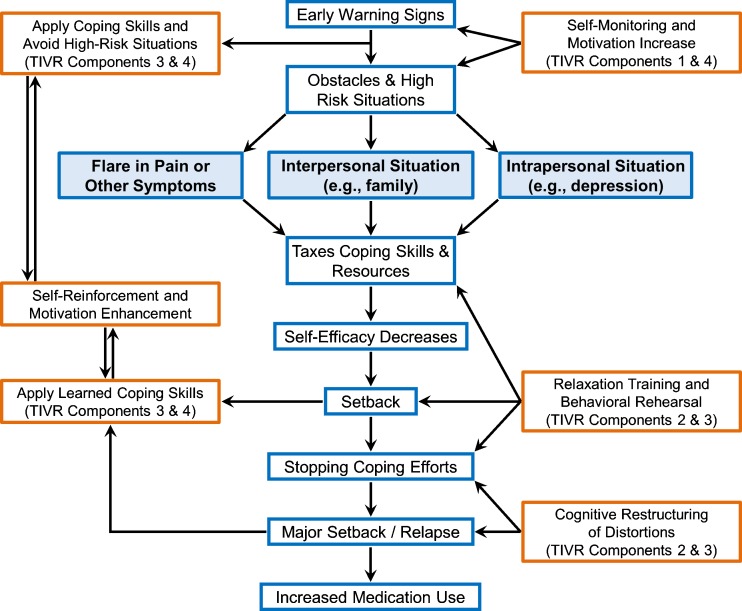
A relapse prevention model of coping with pain: this model depicts the interrelationships between chronic pain, psychosocial stressors, therapy, and coping skills use for chronic pain. Adapted from F. Keefe by M. Naylor [59], reprinted with permission

This specific TIVR regimen consists of four components: (1) automated access to self-monitoring of symptoms, (2) didactic review sessions of coping skills, (3) guided behavioral rehearsals of pain management skills, and (4) personalized encouragement and reinforcement. The relationship between these four components of TIVR and their roles in relapse prevention are outlined in Fig. 1.

**Fig 2:**
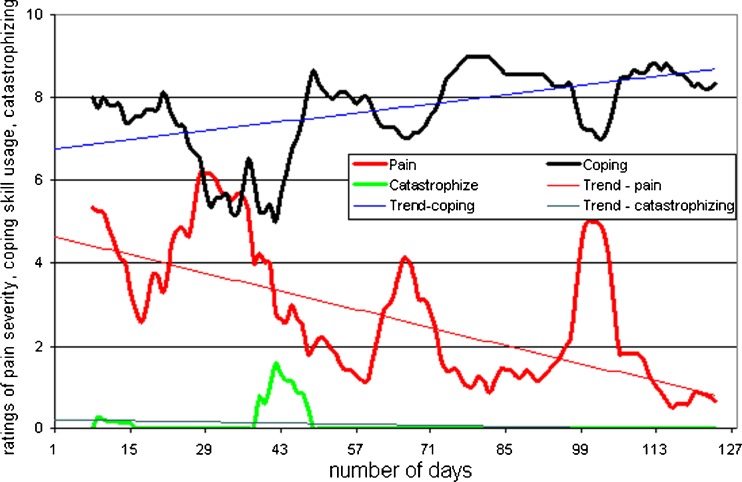
Single subject daily TIVR data: an example of an IVR daily data chart depicting trends in and relationships between pain, coping, and catastrophizing over a 4-month calling period (7-day moving averages). Currently, these charts are utilized by the clinician to monitor patient symptom progression and create feedback messages. In the future, graphs like this might also be sent to patients via email or smartphone applications for visual emphasis of treatment progress

Symptom self-monitoring is comprised of a daily questionnaire to assess levels of pain, mood, and stress, use of pain medications, and a variety of other parameters. Didactic review sessions and guided behavioral rehearsals are offered for eight different coping skills learned previously during group therapy such as relaxation techniques, cognitive skills, activity–rest pacing, and reappraisal of pain. This variety of coping techniques ensures that patients are aware of multiple options for coping with pain, an important part of the relapse prevention model [59, 99]. The guided rehearsals not only remind patients of their coping options but may also enhance patients’ feelings of self-efficacy in their ability to deal with their pain. Self-efficacy may improve because TIVR promotes self-directed treatment by allowing patients to be proactive and monitor the relationships between continued use of coping skills and improvement of pain-related symptoms [44]. It has been suggested that IVR’s ability to help patients set and achieve small, realistic, and incremental goals may contribute to increased self-efficacy [100]. The final component of the TIVR approach (personalized encouragement) consists of monthly messages for each patient recorded by the CBT group therapist. These messages contain a summary of the patient’s daily reports to the TIVR system for the past month, insights into possible relationships between reported pain, negative emotions, and use of coping skills; suggestions for pain management tactics; and verbal encouragement. The treatment efficacy assessments for monthly messages can be easily visualized by compiling the data into a longitudinal chart of symptom progression (Fig. 2).

The relapse prevention model suggests that this regular monthly feedback, which simulates the weekly feedback provided during the initial CBT training, likely helps patients to gauge their progress and recognize successes and problems in coping with difficult situations [101]. The personalized monthly messages may also serve to further strengthen patients’ sense of self-efficacy.

After 4 months of TIVR, patients reported that the method reinforced the skills that they learned during face-to-face CBT, enhanced their motivation to continue using coping skills, and provided structure that helped the new skills become habits [44, 71]. The data showed that patients who were randomized to the TIVR program experienced significant improvements after both 4 and 8 months of CBT as compared to baseline and to control subjects who received CBT but did not participate in TIVR [71]. TIVR subjects also reported decreased use of opioid analgesics [59]. Considered together, these results strongly suggest that TIVR has the ability to extend the treatment gains associated with CBT for coping with chronic pain long after clinical visitations have ceased.

## FUTURE DIRECTIONS AND CONCLUSIONS

Since TIVR was first demonstrated to be an effective adjunct to therapy for chronic pain, a similar system was employed to treat pain and other symptoms in cancer patients [78]. In this study, patients in both an “Automated Telephone Symptom Management” group and a “Nurse-Assisted Symptom Management” control group showed clinically significant improvements in symptom severity. The authors suggested that these specific patient responses could effectively be used to tailor follow-up assessments and interventions to specific patient needs. Another recent pilot study [41] investigated the use of TIVR for relapse prevention following CBT for alcohol use disorders. Automated IVR “self-administered psychotherapy” has proven to be an effective mediator of symptoms of depression [40] and, while not specifically IVR-based, there is evidence that self-administered and minimal-contact therapies are also useful in treating anxiety [102]. In combination with the findings regarding TIVR and chronic pain, these results suggest that TIVR might be successfully applicable to treating a variety of other medical conditions in addition to chronic pain.

Current research is being conducted to test the hypothesis that TIVR without personalized monthly messages from a therapist can be as effective as with monthly messages. If confirmed, the TIVR method would become even more affordable to the general public. Because some patients might benefit from using a more visual interface than that provided by IVR, further investigation is required to determine whether the effectiveness of TIVR could be further improved by adaptation to an internet-based approach. Such an approach could include pre-recorded visual interactions with an actual or simulated therapist, or even just written text presented simultaneously with pre-recorded messages/questions. It would even be possible to incorporate video communication into this type of approach, allowing for both automated and live interactions with a therapist. Adaptation of TIVR to a smartphone application is another possible next step for this emerging technology. This would allow both the visual interactions that are currently achievable via the internet as well as the convenience of accessing the IVR system using a portable, hand-held device.

Another possibility that merits further investigation is whether IVR as a therapeutic intervention is effective enough to actually replace CBT for people who cannot afford therapy or for whom CBT is unavailable. Overall, IVR has so far proven to be an effective, convenient, inexpensive, and reliable method for monitoring symptom severity, tracking treatment progress, and for extending the therapeutic gains of clinical interventions for chronic pain. As technology improves, and as more questionnaires, surveys, and therapeutic interventions are adapted for automated administration, IVR will likely play an increasingly important role in both clinical research and treatment regimens for patients with many different psychological and physiological disorders. We therefore recommend that IVR-based treatment approaches be employed by multidisciplinary clinics and practitioners who treat patients with chronic pain. Further, as IVR-based interventions are clinically beneficial, versatile, and cost-effective; we advise that policymakers endorse insurance coverage of IVR for pain management as part of comprehensive treatment plans.
